# 3D Finite Element Simulation of Graphene Nano-Electro-Mechanical Switches

**DOI:** 10.3390/mi7080143

**Published:** 2016-08-15

**Authors:** Jothiramalingam Kulothungan, Manoharan Muruganathan, Hiroshi Mizuta

**Affiliations:** 1 School of Materials Science, Japan Advanced Institute of Science and Technology, Nomi, Ishikawa 923-1292, Japan; jothi@jaist.ac.jp (J.K.); mizuta@jaist.ac.jp (H.M.); 2 Nanoelectronics and Nanotechnologies Research Group, Faculty of Physical Sciences and Engineering, University of Southampton, Highfield, Southampton SO17 1BJ, UK

**Keywords:** graphene, nanoelectromechanical (NEM) switches, pull-in and pull-out characteristics, von Mises stress

## Abstract

In this paper, we report the finite element method (FEM) simulation of double-clamped graphene nanoelectromechanical (NEM) switches. Pull-in and pull-out characteristics are analyzed for graphene NEM switches with different dimensions and these are consistent with the experimental results. This numerical model is used to study the scaling nature of the graphene NEM switches. We show the possibility of achieving a pull-in voltage as low as 2 V for a 1.5-μm-long and 3-nm-thick nanocrystalline graphene beam NEM switch. In order to study the mechanical reliability of the graphene NEM switches, von Mises stress analysis is carried out. This analysis shows that a thinner graphene beam results in a lower von Mises stress. Moreover, a strong electrostatic force at the beam edges leads to a mechanical deflection at the edges larger than that around the center of the beam, which is consistent with the von Mises stress analysis.

## 1. Introduction

The standby power consumption of conventional complementary metal-oxide semiconductor (CMOS) circuits increases to the dynamic ON state level as they are scaled down to the scale of a few tens of nanometers [1]. On the other hand, nanoelectromechanical (NEM) switches are being investigated because of their promise for future low-power-consumption applications [2,3]. The switching operation of such devices is mainly based on electrostatic actuation, which leads to a very low leakage current and high ON/OFF ratios. They are also expected to achieve abrupt switching with subthreshold swing values less than 60 mV/dec [4]. Furthermore, NEM switches are expected to be robust against external disturbances such as radiation and temperature fluctuations, which makes them ideal for inhospitable environments [5]. On the other hand, the inherently low ON current in comparison with that of CMOS devices and the high pull-in voltage of conventional NEM switches restrict their use as a viable alternative for CMOS circuits and low-power applications [6,7].

Graphene is a two-dimensional material with excellent mechanical stability and electrical conductivity, and a high Young’s modulus of ~1 TPa [8]. These outstanding properties of graphene make it a very promising material for high-performance NEM contact switches. Using graphene as a material for such devices can address some of the problems of conventional NEM switches by providing high reliability and a low pull-in voltage [9,10,11].

In this work, we present a three dimensional (3D) finite element method (FEM) simulation of double-clamped nanocrystalline graphene beam NEM switches. We focus on designing the graphene NEM switch in line with the experimental work [12]. The pull-in and pull-out characteristics from the simulation results are consistent with the experimental results. These models were used to study the double-clamped beam scaling on switching characteristics. After validating the simulation results for the pull-in voltage of the double-clamped graphene beam NEM switch with the experiment results, we studied the von Mises stress to evaluate the reliability of the switch. These results indicated that a longer and thinner graphene beam is more reliable. In addition, we also analyzed the effect of the applied electric field on the double-clamped graphene beam NEM switch.

## 2. Description of the Device Geometry

**Figure 1:**
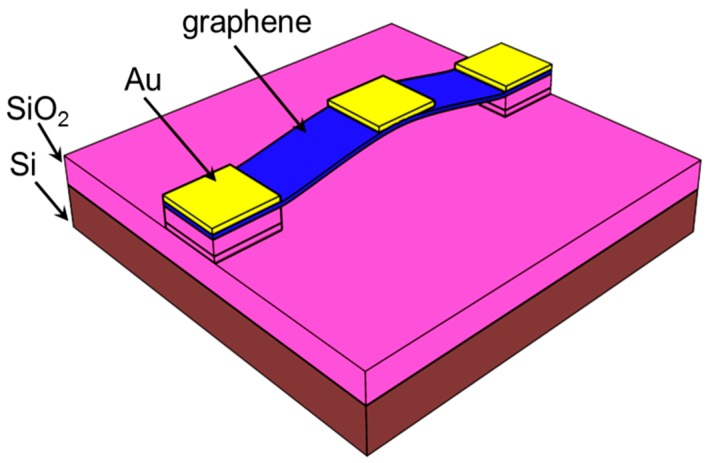
Schematic of double-clamped graphene beam NEM switch with top metal actuation electrode.

In this section, we briefly describe the geometry and operation principles of NEM switches. In our earlier work [12], we experimentally studied the switching operation of nanocrystalline graphene (NCG) beam NEM switches. The NCG was synthesized by direct deposition of NCG on an Si/SiO_2_ substrate using plasma-enhanced chemical vapor deposition (PECVD) [13]. The NCG deposited by PECVD contains both sp^2^- and sp^3^-hybridized carbon atoms. The deposited NCG film is polycrystalline in nature [12]. The polycrystalline nanographene has randomly distributed grain orientation and size [14]. The mechanical behavior of NCG depends on both the grain misorientation and the grain boundary rotation [15]. Moreover, the mechanical strength of NCG depends on the arrangement of the defects in the NCG film [16]. The NCG sheets have almost constant fracture stress and strain, and the fracture strength is independent of the grain size [17]. The polycrystalline NCG sheets have a flaw-insensitive fracture mechanism [18]. Usually, fractures in NCG film originate from the grain boundary and propagate to the rest of the polycrystal [19]. The propagation of the fracture also depends on the grain orientation. Moreover, microcracks in the surfaces are initiated from topological defects in the NCG polycrystal, which coalesce to form a big crack, eventually leading to the breakdown of the NCG film. As NCG is a polycrystalline material, we considered it as an isotropic material in the FEM simulation of the nanocrystalline NEM switches. The device structure and dimensions are adopted from our previous experimental work [12]. We used a Young’s modulus of 860 GPa for graphene in all the FEM simulations as reported by this experimental work. This value is comparable to the reported Young’s modulus of 500 GPa for layered suspended graphene sheets of between 2 and 8 nm thickness [20]. For the FEM simulation of the experimental device, we considered a graphene beam of length *L*, width *W*, and thickness *t*, and a top metal electrode. The schematic representation of the device is shown in Figure 1. The double-clamped graphene beam NEM switch dimensions are detailed in Table 1. For each double-clamped graphene beam NEM switch, the air gap thickness changes owing to the natural buckling of the suspended graphene beam.

The initial air gap of the device is *g*_0_. As the voltage applied between the suspended graphene beam and the top electrode is increased, the resulting electrostatic force balances with the elastic restoring force of the deformed graphene. When the applied voltage reaches a critical point, the electrostatic force overwhelms the restoring force, which causes the graphene beam to be pulled towards the top electrode/fixed element, resulting in closing the switch and thereby leading to a sharp rise in the current flow through the device [21]. When the applied bias voltage is sufficiently reduced, the elastic restoring forces in the deformed active element pull the switch open, and thus the device operates as a volatile switch. A mechanical hysteresis is formed between the pull-in voltage and the pull-out voltage.

## 3. Finite Element Method Simulation Results and Discussion

### 3.1. Graphene NEM Switch Pull-In and Pull-Out Characteristics

**Figure 2:**
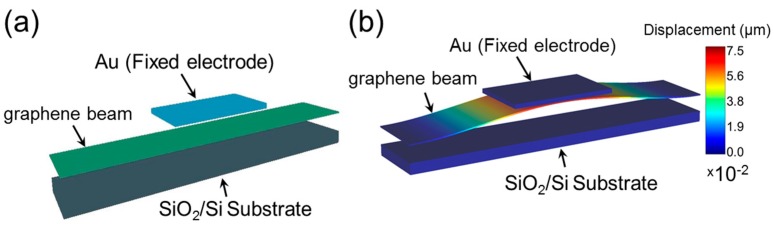
The geometry of NEM switch A. (**a**) Initial structure of double-clamped graphene beam NEM switch with a top metal electrode; (**b**) Pull-in state of the graphene beam; color bar indicates the relative displacement with respect to the initial condition.

The static electrical and mechanical characteristics of the NEM switch were simulated using the FEM-based CAD tool IntelliSuite (8.8.5.1, IntelliSense, Lynnfield, MA, USA) [22]. Figure 1 shows a schematic of our graphene beam NEM switch with a graphene beam connected to electrodes at each end. This graphene beam NEM switch features a metal top gate (actuation electrode), which enables the graphene beam to be pulled onto the gate when a voltage is applied, and then pulled away, disconnecting from the channel when voltage is no longer applied. In order to be consistent with our experimental device structure, we used the device dimensions mentioned in Table 1. Figure 2a shows the initial geometry of NEM switch A used in the FEM simulation. To analyze the pull-in and pull-out characteristics of the NEM switch, the voltage applied between the top electrode and the graphene beam was first increased until pull-in was confirmed and then decreased back to 0 V. Figure 2b illustrates the geometry of the pull-in state of the double-clamped graphene beam NEM switch. The color bar shows the displacement of the graphene beam with respect to the initial position.

**Figure 3:**
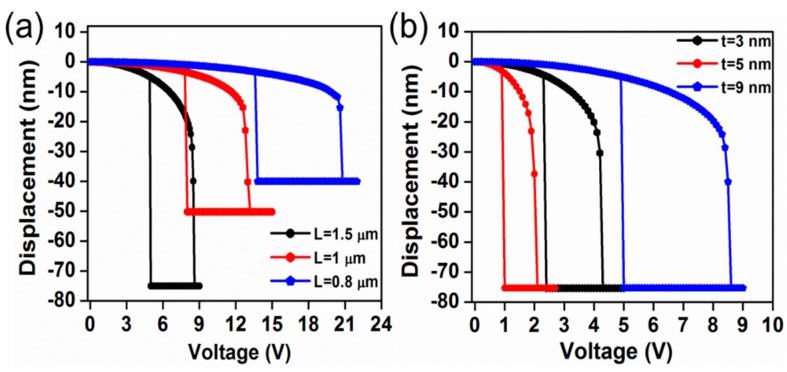
Pull-in and pull-out switching characteristics of the double-clamped graphene beam NEM switches. (**a**) Switching characteristics of graphene beam NEM switches A, B, and C; (**b**) Switching characteristics of graphene beam NEM switch A, with different graphene thicknesses of *t* = 3, 5, and 9 nm.

Figure 3a shows the pull-in/pull-out characteristics obtained for the different graphene beam NEM switches mentioned in Table 1. The pull-in voltages for the graphene beam NEM switches A, B, and C are approximately 8.6, 13.2, and 20.8 V, respectively. The obtained pull-in voltage shown in Figure 3a is in good agreement with the experimentally reported pull-in voltages [13]. In order to clarify the impact of the thickness of the graphene beam, we conducted the FEM simulation for one of our experimental device structures (NEM switch A) with different graphene thicknesses of *t* = 3, 5, and 9 nm. Figure 3b shows the pull-in and pull-out characteristics for the graphene beam with different thicknesses for the voltage applied between the top gate and the graphene beam. When the thickness is reduced, the pull-in voltages are evaluated to be 8.6 V, 4.3 V, and 2.1 V for *t* = 9 nm, 5 nm, and 3 nm, respectively. This result shows a clear dependence of the pull-in voltage on the scaling of the thickness of the suspended graphene beam. Thus, we confirm that the introduction of thickness scaling leads to a reduction of the pull-in voltage.

### 3.2. Von Mises Stress Analysis

The von Mises yield criterion is a general way to estimate the yield of any ductile material, such as metals [23,24]. The mechanical reliability of the graphene beam NEM switch can potentially be improved by properly choosing the switch dimensions. To quantitatively demonstrate the mechanical reliability of the double-clamped graphene beam NEM switch, we compared the maximum von Mises stress exerted along the length of the graphene beam [25,26]. The von Mises stress profile analysis is essential to comprehend the spatial variation of the stress generated on the suspended graphene owing to the applied voltage. A Cartesian coordinate system is used to represent the numerical coordinates on the suspended graphene beam. The stress profile was obtained after the pull-in state was achieved, giving the three-dimensional stress profile for the deformed graphene beam. However, the stress variation along the thickness is constant.

**Figure 4:**
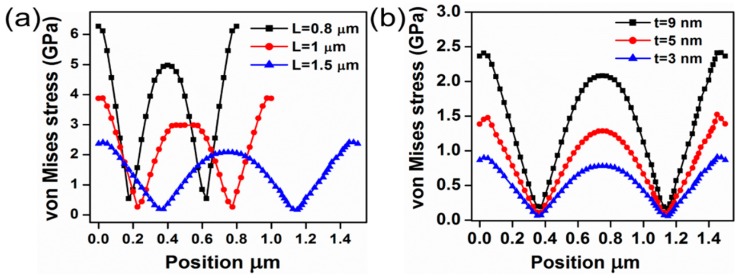
The von Mises stress of double-clamped graphene beam NEM switches. (**a**) von Mises stress of graphene NEM switches A, B, and C; (**b**) von Mises stress of graphene beam NEM switch A, with different graphene thicknesses of *t* = 3, 5, and 9 nm.

Figure 4 shows the von Mises stress for the different graphene beam NEM switches. The von Mises stress reaches the maximum value towards the ends of the graphene beam. When the length of the graphene beam is reduced, the von Mises stress is increased to the maximum value. As evident from Figure 4a, the device with the shortest graphene beam length has the maximum probability of failure. When the thickness of the graphene beam is scaled for the fixed length of the beam, the von Mises stress is reduced as the thickness is reduced. The results suggest that NEM switch A is at least as reliable as NEM switches B and C.

**Figure 5:**
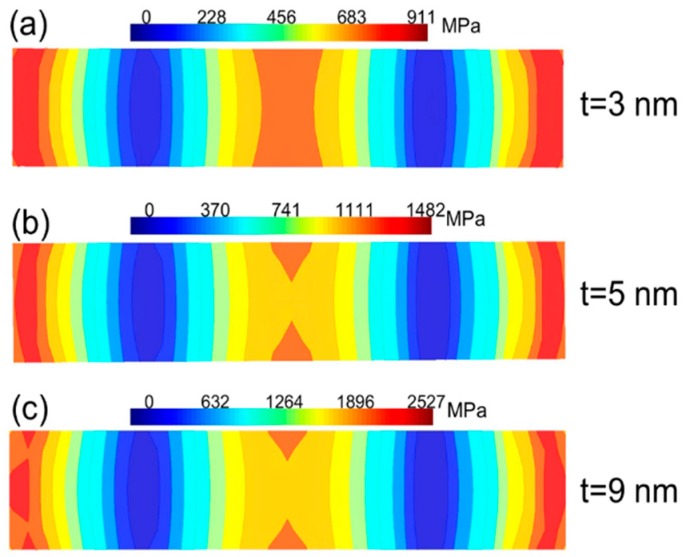
Contour plot (top view of graphene beam) of von Mises stress for the NEM switch A, with different graphene beam thicknesses of *t* = 3, 5, and 9 nm.

NEM switch C has a maximum von Mises stress of 6.2 GPa. When the length of the graphene beam is increased to 1 μm and 1.5 μm, it leads to a decrease in the von Mises stress of 3.8 GPa and 2.3 GPa, respectively. Furthermore, the maximum stress for NEM switch A is 2.5 GPa; when the thickness of the graphene is reduced to 5 nm and 3 nm, the stress is decreased to 1.4 GPa and 0.9 GPa, respectively. Figure 5 illustrates the top view of the von Mises stress contour plot of the graphene beam. It is evident from the contour plot that the von Mises stress is highest nearer to both fixed ends of the beam. The von Mises stress reaches the minimum value between the fixed end of the beam and the center of the beam in the pull-in state. If we examine the von Mises stress across the beam carefully, then we can observe higher stresses at the edges of the graphene beam compared to those at the center of the beam. In order to clarify this point, 3D electric field distributions in the NEM switch were carried out in the pull-in state.

### 3.3. Three-Dimensional Electric Field Distribution and Its Role in Graphene Beam NEM Switch Operation

**Figure 6:**
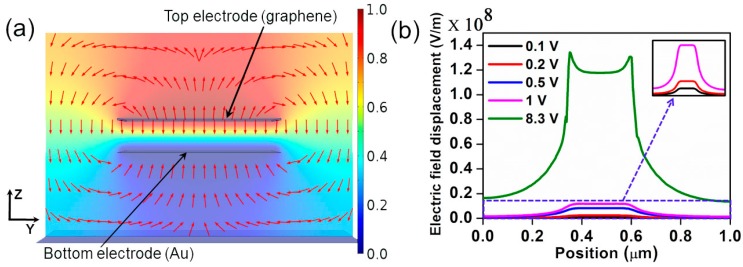
Two-dimensional electric field distribution across the center of the NEM switch. Dimensions of the graphene beam (top electrode) are equivalent to those of NEM switch A. (**a**) The electric field distribution across the switch at the center of the beam. Arrows indicate the electric field direction; (**b**) The electric field strength in the *Z* direction at 5 nm above the bottom electrode at different voltages applied between the bottom and top electrodes. Inset shows zoomed-in version of electric field distribution for lower actuation voltages as indicated by the dashed box; the scale is the same as (**b**).

To analyze the impact of the applied electric field on the double-clamped graphene beam NEM switch, we made the same model in COMSOL Multiphysics (5.1, COMSOL Inc., Burlington, MA, USA) [27]. The NEM switch was built inside a vacuum environment. The model was meshed with triangular mesh elements to reduce the computational complexity. The density of the mesh was varied adaptively in order to study the structural displacement of the graphene beam. In this simulation, the actuation electrode was kept at the bottom and the graphene beam was placed at the top. For the electric field analysis at different voltages, a constant bias of 0 V was applied to the bottom electrode (Au) and the voltage applied at the top electrode (graphene beam) was swept. The potential, *V*, and the electric field, *E*, in the free space can be obtained by solving Poisson’s equation [28]. Figure 6a shows the cross-sectional view of the electric field distribution across the center of the NEM switch for the applied voltage of 1 V to the bottom electrode. The dimensions of the graphene beam are equivalent to those of NEM switch A. Arrows in this plot show the electric field lines directions. At the center of the beam, the electric field lines are distributed vertically. The orientation of the electric field distribution is gradually changed to the horizontal direction towards the edges of the beam. At both edges of the beam, the electric field is distributed more horizontally in the outward direction from the center of the beam.

**Figure 7:**
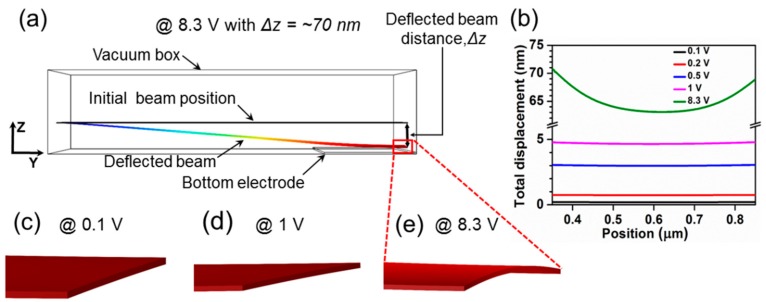
Effect of the beam edge electric field termination at 8.3 V. (**a**) The displacement of the beam at 8.3 V shown as side view; (**b**) The displacement of the beam from initial position at different applied voltages. Birds-eye cross-sectional view of the graphene beam at (**c**) 0.1 V; (**d**) 1 V; and (**e**) 8.3 V.

Figure 6b illustrates the one dimensional (1D) electric field strength in the *Z* direction at 5 nm above the bottom electrode for the different applied voltages. Consistent with Figure 6a, the electric field strength is highly concentrated at the edges of the graphene beam. These results demonstrate that the downward component of the electrostatic force acting on the edges of the graphene beam is higher than that at the center of the beam. In order to analyze this edge field termination effect, mechanical deflection analysis of NEM switch A with a 3 nm graphene thickness was done. Figure 7 shows the displacement of the graphene beam nearer to the pull-in state. If we consider the edge of the graphene beam, then the downward bend of the beam edges is apparent. This is also consistent with a higher von Mises stress at the beam edges.

## 4. Conclusions

In this paper, we have studied the electro-mechanical switching and mechanical reliability of graphene beam NEM switches by FEM simulations. To evaluate the mechanical reliability of a graphene beam NEM switch, we scaled the length and thickness of the graphene beam and studied the von Mises stress for each structure. This analysis showed that the graphene beam NEM switch with a longer length of 1.5 μm and a thickness of 3 nm has a pull-in voltage of 2 V. The electrostatic force concentration at the edges of the graphene beam leads to more mechanical deflection at the edges than at the center of the beam.

## Figures and Tables

**Table 1:** NEM switch dimensions.

NEM Switch	Dimension			Air Gap Thickness (nm)
	Length (μm)	Width (μm)	Thickness (nm)	
A	1.5	0.5	9	75
B	1	0.5	9	50
C	0.8	0.5	9	45
